# Editorial: Advanced Polymer Composite Materials: Processing, Modeling, Properties and Applications II

**DOI:** 10.3390/polym16182650

**Published:** 2024-09-20

**Authors:** Giorgio Luciano, Maurizio Vignolo

**Affiliations:** National Research Council of Italy—Institute of Chemical Sciences and Technologies “Giulio Natta” CNR SCITEC, Via De Marini 6, 16149 Genova, Italy

Building on the success of our first Special Issue, we are pleased to present this second collection dedicated to the multifaceted world of composite materials. The application of these materials continues to span numerous domains of human endeavor, from agriculture and industry to environmental protection, biomedicine, and transportation [1,2,3,4,5,6,7,8,9,10,11,12,13,14,15,16,17,18]. In the face of ongoing energy and climate challenges, the importance of research into polymer-based composite materials has become increasingly evident. Such studies have the potential to profoundly impact our current society and, more critically, shape a sustainable future for generations to come.

This second Special Issue further showcases the evolving landscape of composite materials research, presenting a diverse array of high-quality scientific contributions. The selected articles cover a wide spectrum of topics, from fundamental materials science to practical applications, highlighting the versatility and indispensability of composite materials in our daily lives.

A notable feature of this collection, building upon themes explored in our first issue, is the increased focus on biopolymers, such as chitosan and polylactic acid [6,10]. This reflects the growing trend towards sustainable and environmentally friendly materials. These biopolymer-based composites offer promising solutions for various applications, including water treatment, drug delivery, and biodegradable packaging.

The variety of applications explored in this second issue is truly remarkable and expands upon the foundations laid in our previous collection. We see innovative composite materials being developed for electronic applications, such as flexible antennas and smart windows [8,13], showcasing the potential of these materials in the realm of next-generation technologies. In the field of construction and civil engineering, novel composite reinforcements for concrete structures are presented, offering improved durability and performance [5,14].

Environmental applications continue to be well-represented, with studies on composite materials [2,3,5,10,17]. In the energy sector, we find exciting developments in materials for solar cells and thermal management, addressing crucial challenges in renewable energy and energy efficiency [4].

The breadth of characterization techniques and methodologies employed in these studies, from advanced microscopy to molecular dynamics simulations, further emphasizes the multidisciplinary approach required in modern materials science [2,5].

This second Special Issue not only demonstrates the current state-of-the-art in composite materials research but also highlights the rapid progress being made in the field since our first issue. It points towards future directions and challenges, showing how the landscape of composite materials is continuously evolving. It is our hope that this collection will inspire further interdisciplinary collaborations and innovations, driving the development of next-generation composite materials that can address the complex challenges of our time [14].
